# Response to ad libitum milk allowance by crossbred dairy and dairy–beef calves in an automated feeding system

**DOI:** 10.1093/tas/txad063

**Published:** 2023-07-19

**Authors:** Sara C Arens, Kirsten T Sharpe, Michael M Schutz, Bradley J Heins

**Affiliations:** Department of Animal Science, University of Minnesota, St. Paul, MN 55108, USA; West Central Research and Outreach Center, University of Minnesota, Morris, MN 56267, USA; Department of Animal Science, University of Minnesota, St. Paul, MN 55108, USA; Department of Animal Science, University of Minnesota, St. Paul, MN 55108, USA; West Central Research and Outreach Center, University of Minnesota, Morris, MN 56267, USA

**Keywords:** automated feeder, behavior, calf, crossbreeding, growth, milk allowance

## Abstract

The objective of this study was to compare three-breed rotational crossbred calves sired by Holstein, Jersey, Montbéliarde, Normande, Viking Red, and Limousin bulls with Holstein’s calves fed a high milk allowance for growth, milk consumption, health scores, and profitability in an automated group feeding system. Breed groups were Holstein (*n* = 16), crossbreds of Montbéliarde, Viking Red, and Holstein (*n* = 24), crossbreds of Jersey, Normande, and Viking Red (*n* = 6), and Limousin crossbred beef × dairy (*n* = 45) calves. Calves were randomly assigned within the breed to one of two treatments from September 2019 to June 2020 at the University of Minnesota West Central Research and Outreach Center in Morris, MN. The five breed groups were balanced across the two treatment groups. Treatment groups were fed 8 L/d (8 L) or ad libitum (AL) milk allowance, and calves were introduced to the automated feeder at day 5 and were weaned at 56 d. Milk feeding behaviors (drinking speeds) were collected from the automatic feeding system and analyzed by feeding and breed groups. Body weights were recorded at birth and weekly through weaning. The health scores of calves were recorded twice per week. Variables included in the statistical model for analyses were fixed effects of birthweight, the season of birth, breed group, and treatment group. Calves fed AL had a greater weaning weight (*P* = 0.001; 106.4 kg vs. 91.4 kg) and greater (*P* = 0.001) average daily gain (ADG; 1.11 kg/d vs. 0.87 kg/d) than calves fed 8 L, respectively. The calves fed AL (1,064 mL/min) had a slower drinking speed (*P* = 0.01) than calves fed 8 L (1,467 mL/min). Most breed groups were not different for weaning weight or ADG across the 56 d. Daily milk consumption per calf was lower (*P* = 0.009) for Limousin crossbred calves compared with Holstein and crossbred dairy calves. As expected, AL calves had higher (*P* = 0.001) milk cost ($189.52) than the 8 L calves ($140.71). The average cost per kilogram of gain was similar for calves fed 8 L ($2.89/kg) compared to AL ($3.00/kg) calves. Overall, the Limousin crossbred calves had the least milk cost ($152.75) compared with Holstein ($175.67) calves and Montbéliarde, Viking Red, and Holstein crossbred calves ($177.13). The results from this study found that although feeding calves AL resulted in greater milk consumption and higher cost than 8 L calves, there may be an economic advantage with costs per kilogram of gain to feeding calves ad libitum if increased growth rates are realized.

## Introduction

Rearing of calves in groups has increased in popularity in the dairy industry, even while most calves are still housed individually (Hepola, 2003). With this change, the use of automated, or computer-controlled feeding systems (**AFS**) has become more common in practice on dairy farms to efficiently feed calves housed in groups (Knauer et al., 2017). Computer-controlled AFS originated in Germany in the 1980s and were first exported to Sweden in 1988, followed by Norway in 1989 (Hepola, 2003). The advantages of AFS are to have milk or milk replacer feed to calves at a constant temperature during feeding and to record the daily behavior of calves through the identification of calves with the AFS system (Hepola, 2003). Automated feeding systems also utilize test access which grants calves the opportunity to express natural behaviors, slow milk intake to improve milk digestion and reduce cross-sucking in grouped calves (Khan et al., 2011).

In the past, dairy farmers typically fed calves 4 L of milk per day. Urie et al. (2018) reported the average feeding level for heifer calves was 5.6 L/d. Currently, more farmers are evaluating milk-feeding levels of eight or more liters per day (Heinrichs et al., 2020). However, farmers may be apprehensive about increasing the amount of milk offered to calves due to the potential for increased occurrence of looser manure and increased cost of production (Conneely et al., 2014; Korst et al., 2017; Conboy et al., 2022). Nonetheless, increased milk feeding levels may achieve higher ADG for calves, which may benefit farms with greater milk production during the first lactation. Chester-Jones et al. (2017) reported that preweaning average daily gain (**ADG**) and weaning weight were positively correlated with first-lactation milk production. Davis Rincker et al. (2011) reported that increasing heifer calf weight allowed heifers to achieve breeding weight earlier which may lead to decreased calving age and reduced costs for raising replacement heifers.

A few studies compared the use of an AFS to a manual feeding system, feeding individually housed calves by calf workers at set times per day, on calf growth traits and found no significant differences between the systems (Kung et al., 1997; Sinnott et al., 2021). Kung et al. (1997) reported no difference in milk-replacer intake and Sinnott et al., (2021) reported no difference in weaning weight and ADG for calves fed milk replacer in an AFS system.

While the Holstein (**HO**) breed has been the dominant dairy cattle breed in the United States, some farmers are turning to crossbreeding systems to take advantage of breed complementarity and hybrid vigor (Maltecca et al., 2006). There is little research reporting the effects of crossbreeding on preweaned calves. Dhakal et al. (2013) reported pure Holstein calves had significantly heavier calf birth weights compared to JE and HO crossbred calves and purebred Jersey (**JE**) calves had the lightest birth weight. The results of that study also predicted purebred HO calves to weigh 9.3 ± 0.6 kg more than purebred JE calves at birth. Ware et al. (2015) reported HO calves had great weight gain compared to JE calves (+5.5 kg at 42 d of age and +10.3 kg at 56 d of age). Maltecca et al. (2006) reported on health scores between purebred and crossbred cattle and reported HO calves had higher fecal scores and greater days with scours than crossbred calves sired by JE × HO bulls from HO dams. There were no differences reported for respiratory disease scores between the crossbred or HO calves.

No studies have compared whole milk feeding of HO, crossbred dairy calves, and crossbred dairy–beef calves for growth and profitability on an AFS. Furthermore, extraordinarily little research has compared the preweaning effects of feeding calves ad libitum (**AL**) on an AFS. Therefore, the objective of this study was to evaluate the growth, health, and economics of HO, crossbred dairy calves and Limousin crossbred dairy–beef calves fed alternative milk allowances in an automated group feeding system. Furthermore, the study evaluated cost estimates of calves fed high milk allowances, which is lacking in the literature.

## Materials and Methods

### Experimental Design and Collection of Data

All animal procedures involving animal care and management were approved by the University of Minnesota Institutional Animal Care and Use Committee (#1909-37379A). The study was conducted at the University of Minnesota West Central Research and Outreach Center, Morris, MN dairy, where all calves were born. Data were collected on 46 dairy heifer calves and 45 Limousin × dairy crossbred calves born from September to December 2019 (*n* = 49) and March to May 2020 (*n* = 42). Breed groups of calves were HO (*n* = 16; nine calves for AL and seven calves for 8 L/d), crossbreds composed of Montbéliarde, Viking Red, and HO, (**MVH**; *n* = 24; 10 calves for AL and 14 calves for 8 L/d), crossbreds composed of Normande, JE, and Viking Red (**NJV**; *n* = 6; three per treatment), and Limousin-sired crosses from MVH and NJV cows (*n* = 45; **LH** = 22 heifer calves (12 calves for AL and 10 calves for 8 L/d); **LB** = 23 bull calves (10 calves for AL and 13 calves for 8 L/d). The unbalanced numbers of calves per breed group were because of the sex ratios of Holstein and crossbred dairy calves and the dairy herd was two-thirds crossbreed (mostly MVH crossbreds) and one-third Holstein. Furthermore, Limousin was used for mating 40% of the dairy herd. Calf sex is unknown until birth, and calves that were used survived past 3 d of age for the study, so it can be very difficult to predict sex ratios by breed groups of calves before a study begins. The number of calves varied by breed group; however, they contributed meaningful information for breed group comparisons. A power calculation was conducted with PROC POWER in SAS software before the beginning of data collection for the study. Variables for growth and breed comparisons used in the power analysis (means and SD) were based on a previous study with the same breed groups at the research dairy farm (Bjorklund et al., 2013; Heins and Chester-Jones, 2015; Kienitz et al., 2017; Sharpe and Heins, 2021). The minimal meaningful difference for breed groups was five calves per breed group. An analysis of variance with two treatments with five breed groups was used for growth measurements and the power estimate for 40 calves per treatment with an alpha level of 0.05 was 0.957, which is greater than 0.80, which is commonly used when conducting a power analysis.

### Automated Calf Feeder

Calves were separated from their dams within 12 h of birth and housed in individual pens (Calf-Tel I-Series 22|64 1.83 m pen; Calf-Tel, Hampel Corp.), and fed (3.78 L/d in total) colostrum for the first day of life from their dam and pooled transition milk from day 2 to day 4 of life. The total serum protein of the dairy heifer calves ranged from 4.1 to 8.3, and 70% of the calves had over 5.0 g/dL for total serum protein. Serum proteins were not collected for the Limousin crossbred calves. Calves were grouped and introduced to the Holm and Laue HL 100 automated calf feeder (Holm and Laue GmbH and Co KG, Westerronfeld, Germany) at 5 d of age and alternatingly assigned to one of two feeding treatments: 8 L/d (**8 L**; *n* =47) or ad libitum (**AL**; *n* = 44) of whole milk based on birth order. Two pens of calves were formed during the fall of 2019 and two pens of calves were formed during the spring of 2020. Both 8 L and AL calves were raised together in all pens. Each automated feeding pen had an indoor area of 12.2 × 4.9 m bedded with organic oat straw and access to an outdoor area that measured 10.7 × 4.9 m. Calves were weaned at 56 d of age. Whole milk was fed at an average of 13% of the total solids of pasteurized saleable and nonsaleable whole milk. A Misco refractometer (DD-3 Digital-Dairy Refractometer, Misco, Solon, OH, United States) was used weekly to monitor the total solids in milk. The milk averaged 4.4% fat, 3.5% protein, and 5.6% other solids on an as-fed basis. A majority (>90%) of the milk that was fed was saleable. Texturized calf starter and water were provided the free choice in the automated group feeding pens. Calf starter consisted of organic corn, oats, expelled soybean meal, soybean oil, and minerals. The calf starter was mixed on-site with a portable New Holland 358 Grinder-Mixer at the research dairy and contained (as a percentage of DM) 89.5% DM, 18.5% CP, 19.4% NDF, 5.9% crude fat, 6.2% ash, 1.5% calcium, 0.7% phosphorus, 51% NFC, 47% starch, and 0.13 MJ of ME/kg. Individual starter consumption was not recorded because calves were housed in the automatic milk-feeding pens, where the starter was fed to the whole group. The starter was provided to calves within each pen with a Behlen Country galvanized 3 m poly trough feed bunk (Behlen Mfg., Columbus, NE). Ad libitum access to water was provided within each pen with a Ritchie WaterMatic 100 heated waterer (Ritchie Industries Inc., Conrad, IA).

The Holm and Laue HL100 Calf Feeder was used to feeding both treatments, and each pen of calves had two nipple feeding stations (four stations in total). Only two calves (one per pen) were allowed to drink at any time because the feeder did not allow calves to drink from all four nipple feeding stations at one time. From 5 to 56 d of age, calves in both feeding groups did not have a “ramp-up” or “ramp down” phase. Calves were weaned abruptly at 56 d of age. Although gradual weaning is recommended when feeding high levels of milk (Sweeney et al., 2010; Parsons et al., 2021), facility and labor constraints did not facilitate gradual weaning beyond 56 d for this current study. Calves were allowed to consume 8 L/d or ad libitum from day 5. However, for calves in the 8 L group, calves were allowed to consume 2.4 L per feeding and were not allowed to receive more milk until 2 h later. The 8 L calves were allowed to consume 4 L per half day and a total of 8 L/d. The AL calves were allowed to consume as much milk as the calves chose to per day. If an AL calf consumed 9 L at one feeding, they were not allowed to consume more milk until 4 h later. However, these data were not tracked and only the total milk consumed by calves was recorded. All calves were allowed ad libitum access to the nipple feeding station per day.

All calves were weighed on a digital scale (Tru-Test AP600 Platform with an ID5000 Indicator, Tru-Test Group, Auckland, New Zealand) at birth and at weaning in the barn that housed the automatic calf feeder. Hip heights (Nasco Measuring Stick for Dairy Cattle; Nasco, Fort Atkinson, WI) and heart girths (Coburn Calf Weigh Tape, The Coburn Co., Whitewater, WI) were measured at weaning. Body measurements of calves included birth weight, weaning weight, weaning hip height, weaning heart girth, total gain, and ADG. Total weight gain was calculated as the difference between weaning weight and birth weight. The ADG per calf was the difference between birth weight and weaning weight, divided by 56 d. Feeding behaviors, total milk consumed per calf per visit (L) and drinking speed (mL/min), were collected from the Holm and Laue automated calf feeder each time a calf visited the feeding station. Individual health and hygiene scores were recorded on all calves twice per week. The health scoring method was adapted from McGuirk (2018), and calf health was scored twice per week and used physical indicators on a zero to four scale. A fecal score of zero represented normal, and a score of four represented severe watery diarrhea. The calf hygiene scoring method from Kellermann et al. (2020), scored the belly, side, and rear of a calf with dirtiness scores of one to three.

### Economics

Milk feed cost was calculated by the total amount of milk consumed by each calf multiplied by a default milk price of $0.33/kg of liquid milk and summed for each calf. The majority of the milk fed was saleable, and therefore, is reflected in the higher price. Health treatments were documented on an individual calf basis. Total health cost was the sum of the cost of treatments administered to each calf. Total cost was the sum of milk feed cost and total health cost per calf. The average cost per day was the sum of the total cost per calf divided by the days on the AFS (56 d). The average cost per kilogram of gain was the sum of the total cost divided by the total weight gain for individual calves. Sensitivity analyses were performed to evaluate the effects of changes in milk price on total feed cost and the average cost per kilogram of gain for dairy calves. An alternative milk price of $0.22/kg was used for a sensitivity analysis.

### Statistical Analysis

For the analysis of calf body measurements, milk feeding behaviors, milk cost, health cost and total cost, the fixed effects were breed group, and treatment (8 L vs. AL). Season of birth was a covariable in the statistical model. Data were analyzed using PROC MIXED of SAS 9.4 (SAS Institute, 2018). For the analysis of calf health scores, the fixed effects were the same as the growth and economics data analysis, and the scoring date was a repeated measure; however, PROC GLIMMIX of SAS was used for statistical analysis. The compound symmetry covariance structure was used to account for repeated measures because it resulted in the lowest AIC statistic for model fitting criteria. The calf birth weight was a covariate in the statistical model. All treatment results were reported as least squares means with significance declared at *P* < 0.05. Calf was the experimental unit for all analyses. The design was a randomized block design with adjustments for breed group and season. Preliminary analysis for all traits included pen as a fixed effect in the model, as well as another analysis that included pen as a random effect in the model. Pen was included in all statistical models as a random effect.

## Results and Discussion

For the effect of season, calves born during the spring tended (*P* = 0.10) to have greater weaning weight (100.3 vs. 97.5 kg), total weight gain (60.2 vs. 57.4 kg), and greater hip height (95.9 vs. 95.1 cm). Calves born during the fall tended (*P* = 0.10) to have lower health costs ($2.92 vs. $5.88) per calf compared to calves born during the spring. Furthermore, calves born during the fall tended (*P* = 0.09) to have lower total costs ($163.68 vs. $175.35) per calf and lower cost per day ($2.93 vs. $3.13) compared to calves born during the spring, respectively.

Calves born during the fall had higher (*P* = 0.001) fecal scores (0.70 vs. 0.22), scours scores (1.70 vs. 1.18), and respiratory scores (1.05 vs. 1.01), compared to calves born during the spring. Although statistical differences were observed for health scores were observed, biologically the differences were low, and all calves were healthy during the study.

### 8 L/d vs. Ad Libitum Feeding

Results for body measurements, feeding behaviors, and health and hygiene scores overall treatment groups are in Table 1. The AL calves had higher weaning weight (*P* = 0.001), weaning hip height (*P* = 0.002), weaning heart girth (*P* = 0.002), and total gain (*P* = 0.001) compared to the 8 L calves. Furthermore, the AL calves had higher ADG (1.11 ± 0.04 kg/d; *P* = 0.001) compared to 8 L (0.87 ± 0.04 kg/d) calves. Calves allocated 8 L had a faster drinking speed (1467 ± 171.1 mL/min; *P* = 0.001) than AL calves (1,064 ± 172.2 mL/min). The AL calves had worse fecal scores (0.50 ± 0.06; *P* = 0.05) compared to 8 L (0.41 ± 0.06) calves, suggesting that AL calves had a higher occurrence of looser manure than 8 L calves because of the greater milk intake. Heinrichs and Heinrichs (2011) concluded that the number of days a calf had scours or coughing during the first 4 mo of life had a negative impact on the subsequent first-lactation 305-d mature equivalent and milk, protein, and fat production. Quite possibly, the calves fed AL had looser manure because of the greater consumption of milk fed to the calves that lead to the greater fecal output. Therefore, the higher fecal scores could be associated with greater nutrients consumed from the whole milk, and not necessarily disease.

**Table 1.** Means for body measurements, milk feeding behaviors, health and hygiene scores, and milk cost,and economic analysis of dairy calves by milk treatment during the first 8 wk of life^1^

	8 L/d feeding (*n* = 47)		Ad libitum feeding (*n* = 44)	
Measurement	LSM	SE	LSM	SE
Birth weight, kg	38.9	0.9	39.1	0.9
Weaning weight, kg	91.4^a^	2.8	106.4^b^	2.9
Weaning hip height, cm	94.6^a^	0.9	96.4^b^	0.9
Weaning heart girth, cm	104.3^a^	0.7	108.9^b^	0.8
Total gain, kg	51.3^a^	2.8	66.3^b^	2.9
ADG, kg/d	0.87^a^	0.04	1.11^b^	0.04
Drinking speed, mL/min	1,467^a^	171.1	1,064^b^	172.2
Total milk consumed, L	414.9^a^	12.8	557.2^b^	13.2
Milk consumed per calf, L/d	7.5^a^	0.2	10.1^b^	0.2
Fecal score	0.41^a^	0.06	0.50^b^	0.06
Scours score	1.38	0.05	1.49	0.06
Respiratory score	1.01	0.01	1.05	0.01
General appearance score	1.05	0.02	1.03	0.02
Calf side score	1.03	0.02	1.03	0.02
Calf rear score	1.12	0.05	1.07	0.05
Milk feed cost, $	140.71^a^	4.37	189.52^b^	4.56
Health cost, $	4.15	1.67	4.64	1.74
Total cost, $	144.86^a^	4.80	194.17^b^	5.00
Average cost per gain, $/kg	2.89	0.10	3.00	0.10
Average cost per day, $/d	2.35^a^	0.08	3.16^b^	0.08
Lower milk cost, $	93.81^a^	2.91	126.35^b^	3.04
Lower total cost, $	97.96^a^	3.47	130.99^b^	3.62
Lower cost per gain, $/kg	1.95	0.06	2.02	0.06
Lower cost per day, $/d	1.75^a^	0.06	2.34^b^	0.06

^1^Reported means and SE are based on feeding group averages.

^a,b^Means within a row without common superscripts are different (*P* < 0.05).

There were no statistically significant differences in scores for scours, respiratory, general appearance, belly, side, and rear between the two treatment groups. The differences observed for weaning weight and ADG among treatment groups were similar to previous studies that reported that calves fed AL had great weights and ADG (Jasper and Weary, 2002; Rosenberger et al., 2016; Suarez-Mena et al., 2021). Reardon and Everitt (1972) studied twin, male JE and Friesian × JE calves for the effect of preweaning nutrition on growth rates and carcass composition and reported an increase of 2 kg of carcass weight per every added kilogram of weaning weight. While the 8 L calves had a limited supply of milk at the feeder in the current study compared to the AL calves, the 8 L calves had faster drinking speeds than the AL calves. Appleby et al. (2001) and Borderas et al. (2009) reported calves who consumed more milk had faster drinking speeds. The limited-access calves in those studies spent more time at the feeder which could lead to the conclusion that limiting milk access to calves would render the AFS inefficient. Possibly the 8 L calves in the current study were hungrier and therefore consumed milk at a faster pace than those given AL access. Jensen (2006) reported that calves with restricted access spent less time ingesting milk but they also spent more time at the feeder performing nonnutritive sucking. Additionally, drinking speed may vary immensely based on milk-feeding strategies and calves fed less milk have faster drinking speeds than calves fed more milk (Conboy et al., 2022).

Table 1 also has the means for milk cost, health cost, and economic and sensitivity analyses of treatment groups. Milk cost was higher (*P* = 0.001) for the AL calves compared to 8 L ($189.52 vs. $140.71 per calf, respectively) calves due to the increased amount of milk consumed. There were no differences in health costs for 8 L and AL calves. The AL calves had a higher (*P* = 0.001) total cost per calf ($194.17 vs. $144.86) because of the higher milk feed cost. There was no difference in average cost per gain; however, the average cost per day was higher (*P* = 0.001) for AL calves compared with 8 L calves. When adjustment was made for a lower milk cost in the sensitivity analysis, the differences in total cost, cost per gain, or cost per day for 8 L and AL calves remained unchanged compared to the baseline milk cost. Calf grain intake was not recorded on an individual basis and would be difficult to determine because calves being housed in groups. Therefore, had grain intake been able to be individually recorded, total feed costs could be influenced by both milk and calf grain intake.

Calves in this study were healthy throughout, regardless of feeding level and fed at high milk levels. Other studies also noted an increase in fecal scores for calves that were fed a higher milk diet (Jasper and Weary, 2002; Suarez-Mena et al., 2021), while de Paula Vieira et al. (2008) found no increase in scours compared to calves with lower milk diets. As calves consume more milk, it is expected that their manure will have a more liquid consistency compared to a stool with a firmer texture. However, fecal scores for calves in the current study were low for both treatment groups.

### Breed Groups

The least squares mean for body measurements, milk feeding behaviors, health and hygiene scores, milk cost, health cost, and economics by breed groups are in Table 2. There were no differences in weaning weight and total gain among the breed groups. The HO calves had lower ADG (*P* = 0.01) compared with the LB calves (0.93 vs. 1.07, respectively). The differences observed in ADG of HO and crossbred dairy calves in the current study were contrary to other studies that reported on HO and crossbred calves (Ware et al., 2015). Ware et al. (2015) reported that HO calves had higher body weight gain compared to Jersey × HO crossbred calves. However, that study reported on birth weight and growth through 56 d of age for JE crossbred calves, and the Jersey breed has smaller calves and will moderate calf size in a crossbreeding system (Heins et al., 2010; Pereira et al., 2022). Bjorklund et al. (2013) reported that HO calves had similar weaning weight and ADG compared to MVH calves (0.69 kg/d for HO and 0.65 kg/d for MVH), which was similar to the reported ADG in the current study. However, the calves in Bjorklund et al. (2013) were fed 4 L per day, which limited the growth of calves. Very few studies have reported on the health scores of crossbred calves and HO calves. Maltecca et al. (2006) reported HO calves tended to have higher fecal scores than crossbred calves, but respiratory scores were not different for HO calves and crossbred calves.

**Table 2.** Least squares mean and standard errors of means for body measurements, milk feeding behaviors, health and hygiene scores, milk cost, health cost, and economic and sensitivity analysis of dairy calves for breed groups during the first 8 wk of life.

	Holstein (*n* = 16)		MVH crossbreds^1^ (*n* = 24)		NJV crossbreds^2^ (*n* = 6)		Limousin-crossbred dairy–beef heifer (*n* = 22)^3^		Limousin-crossbred dairy–beef bull (*n* = 23)^3^	
Measurement	LSM	SE	LSM	SE	LSM	SE	LSM	SE	LSM	SE
Birth weight, kg	37.7^bc^	1.4	40.6^b^	1.2	33.5^c^	2.3	40.1^b^	1.2	43.9^a^	1.2
Weaning weight, kg	96.9	3.4	99.3	3.1	95.3	4.7	99.9	3.2	103.0	3.2
Weaning hip height, cm	96.4^ab^	1.1	97.2^a^	1.0	95.4^abc^	1.5	94.5^bc^	1.0	94.1^c^	1.0
Weaning heart girth, cm	105.5	2.1	106.9	0.9	105.5	2.1	106.9	1.0	107.1	1.0
Total gain, kg	56.8	3.4	59.2	3.1	55.2	4.7	59.9	3.2	62.9	3.2
ADG, kg/d	0.93^b^	0.04	1.02^ab^	0.03	0.94^ab^	0.08	1.02^ab^	0.05	1.07^a^	0.05
Drinking speed, mL/min	1,117	183	1,299	177	1,263	211	1,313	178	1,337	179
Total milk consumed, L	520.1^a^	20.9	518.8^a^	16.9	505.6^ab^	34.5	439.3^b^	17.8	446.3^b^	17.9
Milk consumed per calf, L/d	9.5^a^	0.4	9.4^a^	0.3	9.2^ab^	0.6	8.0^b^	0.3	8.1^b^	0.3
Fecal score	0.52	0.10	0.32	0.08	0.57	0.14	0.38	0.08	0.50	0.08
Scours score	1.51	0.09	1.29	0.07	1.56	0.14	1.36	0.08	1.47	0.07
Respiratory score	1.03	0.02	1.01	0.01	1.04	0.03	1.05	0.02	1.02	0.02
General appearance score	1.07	0.03	1.02	0.02	1.02	0.05	1.06	0.02	1.02	0.02
Calf side score	1.05	0.03	1.04	0.03	1.02	0.03	1.01	0.02	1.01	0.02
Calf rear score	1.09	0.07	1.04	0.06	1.11	0.08	1.12	0.05	1.16	0.06
Milk feed cost, $	175.67^a^	7.10	177.13^a^	5.83	167.31^ab^	11.32	149.99^b^	6.14	155.50^b^	5.79
Health cost, $	4.27	2.72	2.47	2.23	0.08	4.33	8.10	2.34	7.06	2.21
Total cost, $	179.94^a^	7.79	179.60^a^	6.41	167.39^ab^	12.34	158.08^b^	6.74	162.56^b^	6.36
Average cost per gain, $/kg	3.19^a^	0.15	3.00^a^	0.13	3.16^a^	0.23	2.72^ab^	0.13	2.66^b^	0.13
Average cost per day, $/d	2.93^ab^	0.12	2.95^a^	0.10	2.79^ab^	0.19	2.50^b^	0.10	2.59^b^	0.10
Lower milk cost, $	117.11^a^	4.73	118.09^a^	3.89	111.54^ab^	7.55	99.99^b^	4.09	103.67^b^	3.85
Lower total cost, $	121.39^ab^	5.64	120.55^a^	4.63	111.62^ab^	8.98	108.09^b^	4.87	110.72^b^	4.60
Lower cost per gain, $/kg	2.14^a^	0.11	2.01^a^	0.09	2.10^ab^	0.17	1.86^ab^	0.09	1.81^b^	0.09
Lower cost per day, $/d	2.17^ab^	0.10	2.15^a^	0.08	2.00^ab^	0.16	1.93^b^	0.09	1.98^b^	0.08

^a–d^Means within a row without common superscripts are different at *P* < 0.05.

^1^MVH = crossbreds of Montbéliarde, Holstein, and Viking Red.

^2^NJV = crossbreds of Normande, Jersey, and Viking Red.

^3^Limousin-sired crossbreds from MVH and NJV cows.

The Limousin crossbred calves had lower milk consumption (439.3 ± 17.8 L for heifers and 446.3 ± 17.9 L for bulls) compared to the other breed groups. Notably, the Limousin crossbred calves had the lowest milk consumption while having similar weaning weight and ADG compared to the other breed groups, which may be an indicator of heterosis for feed efficiency of beef and dairy crossbreds (Basiel and Felix, 2022; Jaborek et al, 2023). Similar to the current study, Vestergaard et al., (2019) reported that ADG was similar for Limousine crossbred heifer and bulls compared to HO bull calves.

The economic analysis within breeds is also shown in Table 2. Limousin crossbred calves consumed less milk and consequently had the lowest (*P* = 0.009) milk feed cost compared to HO and MVH. Health costs were not different among the breeds due to the low incidence of disease observed during the study. Total cost, therefore, was lowest (*P* = 0.01) in the Limousin crossbred calves compared to the MVH calves and numerically the lowest total cost compared to HO and NJV calves. Meanwhile, the average cost per gain for Limousin bull calves was the lowest (*P* = 0.03) compared to the other breed groups and the NJV had numerically the highest cost per gain. The same trend also occurred in the average cost per day; however, the MVH calves had numerically the highest cost per day. Few studies have compared beef × dairy crossbred calves compared to HO calves for economics. Recently, McCabe et al. (2022) reported beef × dairy crossbred calves sold for $3.25/kg compared with $2.44/kg for HO calves from online auction services in the United States. A deficiency of many calf studies is that all costs from milk, grain, and health are not accounted for. Grain intake was not recorded in the current study. However, feeding of milk and grain is the main contributor to the increase costs of feeding dairy calves. Future research should explore the economics of dairy × beef calves during the preweaning period (Felix et al., 2023).

Khalili et al. (1992) compared three different milk allocation schedules (2 to 4 L/d) with Friesian × Zebu crossbred dairy calves and reported live-weight gain was significantly higher for calves provided the most milk (252 L total over 24 wk.) throughout the study, compared to the alternate, lower milk allowance treatments (134 L total). Bjorklund et al. (2013) reported on weaning age and milk consumption in group-fed calves with HO, MVH, and NJV dairy calves. Similar to the current study, the NJV calves had the lowest birth weight, and the HO and HMS calves had similar ADG, but HO calves had a higher ADG than NJV calves. Furthermore, Groenendijk et al. (2018) compared AL milk allocation with low allowance (10% initial live weight) and high allowance (HA; 20% initial live weight) using New Zealand crossbred HO × Jersey dairy calves. The AL calves had the greatest ADG preweaning whereas the low allowance calves had the lowest ADG preweaning. Recently, Basiel and Felix (2022) reported that crossbred cattle sired by beef bulls had greater ADG and converted feed to gain more efficiently than pure dairy cattle. Perhaps, the Limousin crossbred beef × dairy calves in this study also converted milk and grain to grow more efficiently than crossbred dairy calves. Additional research should be conducted that evaluates crossbred beef × dairy calves from birth to weaning for growth and efficiency.

## Conclusion

Increasing the quantity of milk leads to an increase in body weight which may have a positive effect on future milk production and reproduction. The current study found that calves fed AL had higher ADG, but higher total costs per calf because of increased milk intake. However, the average cost per gain of calves was similar to calves fed AL or 8 L per day. Health scores of calves were similar among feeding groups, indicating calf health is maintained when feeding higher levels of milk. However, calves fed a higher milk allowance may need to be weaned at greater than 8 wk of age or weaned gradually in a step-down approach after 56 d to maintain calf growth and negate any benefit of providing calves with a higher milk allowance. This study also saw no difference in the health of the calves with increased milk allowance. Both the positive effect of increased weaning weight and decreased health cost may potentially counteract the increased production costs associated with an increased milk allowance for calves. However, more research is needed with different breeds and crossbreds fed different milk allowances to determine if weight gain offsets the cost of raising calves.
